# Naringenin Inhibits Enterotoxigenic *Escherichia coli*-Induced Ferroptosis via Targeting HSP90 in IPEC-J2 Cells

**DOI:** 10.3390/antiox14080914

**Published:** 2025-07-25

**Authors:** Pengxin Jiang, Kangping Liu, Yanan Cui, Puyu Liu, Xutao Wang, Zijuan Hou, Jiamei Cui, Ning Chen, Jinghui Fan, Jianguo Li, Yuzhu Zuo, Yan Li

**Affiliations:** 1 College of Veterinary Medicine, Hebei Agricultural University, Baoding 071001, China; 20222200565@pgs.hebau.edu.cn (P.J.); liukangping@hebau.edu.cn (K.L.); cuiyn@pgs.hebau.edu.cn (Y.C.); 20241200140@pgs.hebau.edu.cn (P.L.); 16682089805@163.com (X.W.); 20232200594@pgs.hebau.edu.cn (Z.H.); 20232200593@pgs.hebau.edu.cn (J.C.); 20237201634@pgs.hebau.edu.cn (N.C.); dyfjh@hebau.edu.cn (J.F.); 2 College of Animal Science and Technology, Hebei Agricultural University, Baoding 071001, China; jgli@hebau.edu.cn

**Keywords:** naringenin, HSP90, ferroptosis, ETEC, oxidative damage

## Abstract

Enterotoxigenic *Escherichia coli* (ETEC) leads to severe diarrhea in piglets. Naringenin (Nar), a natural flavonoid compound, is known for its antibacterial and anti-antioxidant properties. However, the protective effects of Nar against ETEC-induced diarrhea have not been reported yet. This study investigated the protective mechanisms of Nar against ETEC infection in porcine intestinal epithelial cells (IPEC-J2). ETEC infection induced oxidative stress and ferroptosis in IPEC-J2 cells by elevating intracellular iron content and ROS accumulation, increasing MDA levels, downregulating SOD activity and GPX4 expression, and upregulating the transcription of CHAC1 and SLC7A11. In contrast, Nar suppressed ETEC-induced ferroptosis of IPEC-J2 cells by inhibiting the SLC7A11/GPX4 pathway. Specifically, Nar mitigated mitochondrial damage, reduced intracellular iron levels and ROS accumulation, and ultimately reversed the oxidative stress. Network pharmacology and molecular docking identified heat-shock protein 90 (HSP90) as a potential target of Nar. Overexpression and knockdown experiments revealed that ETEC-induced ferroptosis was mediated by upregulation of HSP90, while the protective effects of Nar against ETEC-induced ferroptosis were dependent on the downregulation of HSP90. In conclusion, Nar targets host HSP90 to protect IPEC-J2 cells from ferroptosis caused by ETEC infection. This study demonstrates that Nar is a potent antioxidant natural compound with potential for preventing ETEC-induced intestinal damage.

## 1. Introduction

Enterotoxigenic *Escherichia coli* (ETEC) is a key bacterial pathogen causing postweaning diarrhea (PWD) in piglets. ETEC infection greatly increases the morbidity and mortality of piglets, causing substantial economic losses in the pig industry [1]. ETEC colonizes the surface of intestinal epithelial cells through various types of fimbrial adhesins, such as F4, F5, F6, F18, and F41, and releases enterotoxins that lead to electrolyte imbalance and water efflux of the intestinal cells, ultimately leading to diarrhea which is accompanied by gut barrier disruption [2]. Previous studies have revealed that ETEC induces intestinal oxidative stress in piglets, including increased levels of 8-OHdG and malondialdehyde (MDA) in the duodenum and jejunum [3]. Oxidative stress is thought to be highly coordinated with gut barrier function. On one hand, oxidative stress disrupts epithelial tight junctions by regulating the redistribution of tight junction proteins, namely occludin, zonula occludens-1 (ZO-1), E-cadherin, and β-catenin [4]. On the other hand, oxidative stress also induces apoptosis of epithelial cells, which is involved in the integrity of the intestinal physical barrier [5]. It has been reported that ETEC infection causes Caspase-8-dependent apoptosis in the jejunum of piglets [6].

Ferroptosis, a type of programmed cell death associated with oxidative stress, is characterized by iron-dependent accumulation of lipid peroxides and reactive oxygen species (ROS) [7]. Elevated iron levels play a pivotal role in ferroptosis by catalyzing the Fenton reaction with hydrogen peroxide, generating hydroxyl radicals. These radicals drive ROS accumulation, lipid peroxidation, and the formation of cytotoxic oxidative byproducts such as MDA, ultimately leading to cell death [8]. Glutathione peroxidase 4 (GPX4), a key protein regulator of ferroptosis, protects cells from oxidative damage by catalyzing the reduction in lipid peroxides to non-toxic alcohols, and its inhibition or depletion leads to ferroptosis through the accumulation of cytotoxic lipid peroxides [8]. In many intestinal diseases, such as colorectal cancer, intestinal ischemia injury, and inflammatory bowel disease, intestinal cells display GPX4 inhibition and glutathione (GSH) depletion, resulting in the accumulation of iron and ROS and thus causing ferroptosis [9]. However, it remains unclear whether ferroptosis is associated with ETEC-induced diarrhea and porcine epithelial cell damage.

Currently, ETEC inactivated vaccines, live attenuated vaccines, and genetically engineered vaccines only cover a limited number of fimbriae or enterotoxins, and thus cannot provide effective protection [2,10,11]. Simultaneously, due to antimicrobial resistance, the use of antibiotics in food animals is limited in many countries. Naringenin (Nar) is a natural flavonoid that is widely found in citrus fruits and medicinal plants, exhibits pharmacological activities, including antibacterial, anti-inflammatory, and antioxidant activities, as well as the regulation of lipid metabolism [12,13]. Vikram et al. revealed that Nar inhibits the growth of *E. coli* by disrupting biofilm formation and the AI-2-dependent bacterial quorum sensing signaling pathway [14]. Other studies have demonstrated that Nar plays a vital role in protecting gut barrier function, mainly by attenuating oxidative stress or ferroptosis in radiation-induced intestinal injury and intestinal ischemia/reperfusion [15,16]. However, the potential role of Nar in alleviating ETEC-induced gut barrier disruption has not yet been explored.

Taken together, we hypothesize that Nar may protect the porcine intestinal epithelial cells against ETEC infection-induced oxidative stress by inhibiting ferroptosis. In this study, we verified that ETEC infection induces ferroptosis in porcine intestinal epithelial cells (IPEC-J2 cells) and further investigated the mechanism by which Nar inhibits ETEC infection-induced ferroptosis. Our study shows that Nar is an active natural antioxidant compound that provides a potential strategy for the prevention and treatment of ETEC-induced diarrhea in animals.

## 2. Materials and Methods

### 2.1. Bacterial Strains and Cell Treatments

The ETEC F41 strain was obtained from the National Center for Veterinary Culture Collection (Beijing, China). The IPEC-J2 cell line was obtained from Kobai Biological Company (Nanjing, China). IPEC-J2 cells were seeded in six-well plates and cultured in DMEM (Solarbio, Beijing, China), supplemented with 0.1 mg/mL streptomycin (Solarbio, China), 100 U/mL penicillin (Solarbio, China), and 10% fetal bovine serum (FBS) (Gibco, Waltham, MA, USA) at 37 °C with 5% CO_2_. When the cells reached 90% confluence, the culture medium was replaced with Opti-MEM (Gibco, USA). The cells were infected with ETEC F41 at a multiplicity of infection (MOI) of 400 and then incubated at 37 °C with 5% CO_2_ for 1 h. The supernatant was discarded, and the cells were rinsed three times with sterile PBS, cultured in DMEM for another 17 h, and collected as ETEC-infected samples (18 hpi). To inhibit ferroptosis with either a ferroptosis inhibitor or Nar, the supernatant was replaced with DMEM medium supplemented with 10 μmol/L Ferrostatin-1 (Fer-1; MedChemExpress, Monmouth junction, NJ, USA) or 50 μmol/L Nar (Aladdin, Shanghai, China) after 1 h of ETEC infection, followed by incubation for 17 h. Uninfected wild-type IPEC-J2 cells were used as negative controls (NC). For the ferroptosis activator group, uninfected cells were cultured in DMEM medium supplemented with 1 μmol/L Erastin (Era) (MedChemExpress, USA) for 18 h. Each treatment was performed in triplicate.

### 2.2. RNA Isolation and Quantitative Real-Time PCR (qRT-PCR)

The cultured cells were scraped with a cell scraper. The total RNA of the cells or homogenized tissues was extracted using Trizol (Invitrogen, Waltham, MA, USA), and reverse-transcribed into cDNA using the HiScript^®^ III RT SuperMix for qPCR (+gDNA wiper) kit (Vazyme, Nanjing, China) according to the manufacturer’s instructions. Quantitative real-time PCR was performed using the Taq Pro Universal SYBR qRT-PCR Master Mix (Vazyme, China) via the Eppendorf Real-time PCR system (Germany). The primer sequences used to amplify the ferroptosis-associated genes are listed in Table 1. The relative mRNA levels of the genes were analyzed using the 2^−ΔΔCt^ method, and GAPDH was used as an internal control. All samples were run in triplicate.

### 2.3. Western Blot

Cell samples were washed three times with PBS and detached with 0.05% trypsin (Solarbio, China). RIPA Lysis and Extraction Buffer (Solarbio, China) were used for cell lysis and total protein extraction following the manufacturer’s instructions. After centrifugation, the concentration of protein in the supernatant was assessed with a BCA kit (Solarbio, China). Total cellular proteins were separated using 10% SDS-PAGE and transferred onto PVDF membranes. The membrane was blocked with a 5% blocking solution for 1.5 h at room temperature and then probed with the primary antibody at 4 °C overnight. The primary antibodies include GPX4 (Abmart, Shanghai, China, 1:1000 dilution) and Tubulin (Bioss, Beijing, China, 1:2000 dilution). Tubulin was used as a loading control. After washing with TBST three times, the membrane was incubated with a secondary antibody (Goat anti-Rabbit IgG-HRP Antibody, Absin, Shanghai, China, 1:5000 dilution) at room temperature for 1.5 h. After incubation with secondary antibodies, the immunoblots were developed with WesternBright ECL HRP substrate (Solarbio, China) and imaged with a GelView9000 Lite (Bltlux, Guangzhou, China). Protein quantification was performed with ImageJ 1.8.0 software.

### 2.4. Detection of Intracellular ROS

IPEC-J2 cells were cultured and subjected to relevant treatments in 6-well plates. To inhibit ferroptosis using either a ferroptosis inhibitor or Nar, the supernatant was replaced with DMEM medium supplemented with 10 μmol/L Ferrostatin-1 (Fer-1; MedChemExpress, USA) or 50 μmol/L Nar (Aladdin, Shanghai, China) after 1 h of ETEC infection, followed by incubation for 17 h. For the ferroptosis activator group, uninfected cells were cultured in DMEM medium supplemented with 1 μmol/L Erastin (Era) (MedChemExpress, USA) for 18 h. When the cells reached 90% confluence, the supernatant was discarded, and the cells were rinsed three times with 1 × PBS. The cells were treated with 1 mL of 10 μmol/L intracellular ROS probe DCFH-DA (Beyotime, Shanghai, China) at 37 °C for 20 min. Then, the cells were rinsed three times with 1 × PBS, followed by incubation in 1 mL of Hoechst 33258 stain solution (Servicebio, Wuhan, China) at 37 °C for 10 min. After another rinse with 1 × PBS, the epifluorescence signals were immediately monitored by fluorescence microscopy (Zeiss, Oberkochen, Germany).

### 2.5. Assessment of Intracellular and Serum Superoxide Dismutase (SOD)/MDA Levels

The IPEC-J2 cells were collected from the six-well plate using a cell scraper, washed, and resuspended in 1 mL of 1 × PBS. The cells were then disrupted by ultrasonication. Sonicated IPEC-J2 cells were subjected to assessment of SOD and MDA levels using the Superoxide Dismutase Assay Kit (Njjcbio, Nanjing, China) and the Malondialdehyde Assay Kit (Njjcbio, China) following the manufacturer’s instructions. The optical density (OD) values of SOD and MDA were measured using a microplate reader (Gene Company Limited, Hong Kong, China) at 450 nm and 532 nm, respectively.

### 2.6. Measurement of Intracellular Total Iron

The IPEC-J2 cells were collected using a cell scraper from the six-well plate, washed, and resuspended in 1 mL of 1 × PBS. Then, the cells were disrupted by ultrasonication. The total iron content was measured using the Cell Iron Content Assay Kit (Solarbio, China) according to the manufacturer’s instructions. The OD value at 510 nm was measured using a microplate reader (Gene Company Limited, China) to assess the iron level.

Intracellular ferrous iron (Fe^2+^) was detected using the epifluorescent probe FerroOrange (Dojindo Laboratories, Kumamoto Prefecture, Japan). For live cell fluorescence imaging, IPEC-J2 cells were seeded on a cell culture imaging dish (Solarbio, China) and cultured at 37 °C with 5% CO_2_ overnight. When the cells reached 90% confluency, ETEC F41 was used to infect the cells at an MOI of 400. The supernatant was discarded, and the cells were washed three times with 1 × PBS. Then, the cells were treated with FerroOrange at a concentration of 1 μmol/L, followed by incubation at 37 °C with 5% CO_2_ for 30 min. The images were visualized using fluorescence microscopy (Zeiss, Germany) with an excitation wavelength of 543 nm and an emission wavelength of 580 nm.

### 2.7. Transmission Electron Microscopy (TEM)

Cultured cells in a 6-well plate were prefixed in 2.5% glutaraldehyde fixative solution (Servicebio, Wuhan, China) for 2 h in the dark at room temperature. The cells were quickly scraped off the plate with a cell scraper and resuspended in 2.5% glutaraldehyde fixative solution for overnight fixation at 4 °C in the dark. After post-fixation in 1% osmium tetroxide for 2 h at room temperature, the samples were washed three times with PBS. The samples were dehydrated using gradient alcohol solutions. Subsequently, the samples were infiltrated with an acetone and Embed-812 resin mixture (*v*/*v* = 1/1) overnight and then embedded in pure Embed-812 resin overnight. Sections (60–80 nm) were cut using the EM UC7 Ultra-thin Slicer (Leica, Wetzlar, Germany), stained with dioxyuranium acetate and lead citrate, and observed using the Transmission Electron Microscope HT7800 (HITACHI, Tokyo, Japan).

### 2.8. Network Pharmacology and Molecular Docking

Therapeutic targets of Nar were identified in the TCMSP database (https://old.tcmsp-e.com/tcmsp.php (accessed on 18 March 2024)). Genes associated with piglet diarrhea were acquired from the Genecards database (https://www.genecards.org/ (accessed on 18 March 2024)) and the Comparative Toxicogenomics Database (CTD). The intersection of potential targets for Nar and piglet diarrhea was analyzed using Cytoscape 3.9.1 software. The 3D structure of Nar was obtained from the PubChem database (https://pubchem.ncbi. nlm.nih.gov (accessed on 18 March 2024)), and the corresponding 3D structures of the target proteins were downloaded from the PDB database (https://www.rcsb.org (accessed on 18 March 2024)). AutoDock v4.2.6 software was used for molecular docking and binding energy prediction.

### 2.9. Plasmid Construction and Transfection

Heat-Shock Protein 90 (HSP90) was cloned into the pcDNA3.1 eukaryotic overexpression vector using XhoI and KpnI restriction sites (pcDNA3.1- OE HSP90). The HSP90 siRNA (F: GCAAGAUCAUGAAGGACAUTT; R: AUGUCCUUCAUGAUCUUGCTT) and the corresponding scrambled negative control (NC) were synthesized by GenePharma (Suzhou, China). The IPEC-J2 cells were seeded in six-well plates until they reached 50% confluency. The culture medium was then replaced with opti-MEM, and the cells were transfected with HSP90 overexpression plasmid pcDNA3.1-OE HSP90 (HSP90-OE), empty pcDNA3.1 vector (HSP90-OE NC), HSP90 siRNA (HSP90-KD), or the corresponding scrambled siRNA control (Scramble) using Lipofectamine 2000 (Thermo Fisher Scientific, Waltham, MA, USA), respectively. Six hours after transfection, the culture medium was changed back to DMEM for cell growth. When the cells reached full confluency, they were washed three times with PBS. Total RNA was extracted using Trizol reagent (Invitrogen, USA), and reverse-transcribed into cDNA using the HiScript^®^ III RT SuperMix for qPCR (+gDNA wiper) kit (Vazyme, China).

### 2.10. Statistical Analysis

All experimental data were collected in triplicate. Statistical analysis was performed by one-way ANOVA using GraphPad Prism 8.0 software. Data are presented as mean ± SD. *p* < 0.05 was considered statistically significant. * *p* < 0.05, ** *p* < 0.01, *** *p* < 0.001.

## 3. Results

### 3.1. Infection with ETEC Induces Oxidative Stress in IPEC-J2 Cells

**Figure 1:**
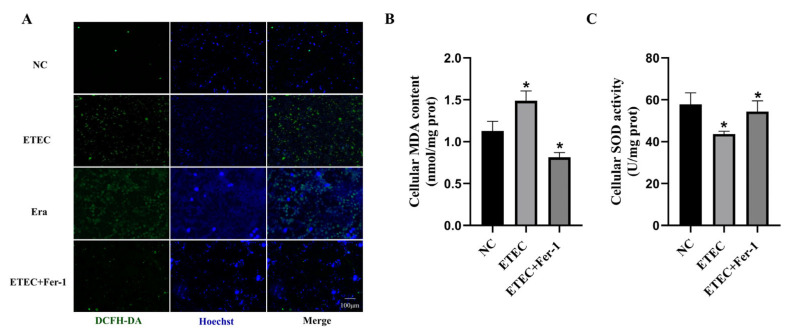
Infection with ETEC induces oxidative stress in IPEC-J2 cells. (**A**) Epifluorescence staining of intracellular ROS (DCFH-DA, green) in wild-type IPEC-J2 cells (NC), ETEC infection (400 MOI), IPEC-J2 cells exposed to 1 μmol/L ferroptosis inducer Era (Era), or both ETEC and 10 μmol/L ferroptosis inhibitor Fer-1 (ETEC + Fer-1). Nuclei were stained by Hoechst (blue). Scale bars:100 μm. (**B**,**C**) MDA and SOD accumulation in wild-type IPEC-J2 cells, or IPEC-J2 cells treated with ETEC or ETEC + Fer-1. Data are expressed as the mean ± SD. * *p* < 0.05.

Intracellular ROS were probed using DCFH-DA to assess oxidative damage of IPEC-J2 cells after ETEC infection. Compared with the wild-type cells, the green fluorescence intensity of ROS sensor DCFH-DA was significantly enhanced due to ETEC infection, consistent with the effects of the ferroptosis inducer Era (Figure 1A). Meanwhile, treatment with the ferroptosis inhibitor Fer-1 greatly reduced the intracellular ROS generation induced by ETEC infection (Figure 1A). At the same time, ETEC infection significantly increased MDA levels and decreased SOD activity in cells, effects that were mitigated by Fer-1 treatment (Figure 1B,C). These results indicate that ETEC infection induces oxidative stress in IPEC-J2 cells via ferroptosis.

### 3.2. ETEC Infection Induces Ferroptosis of IPEC-J2 Cells

**Figure 2:**
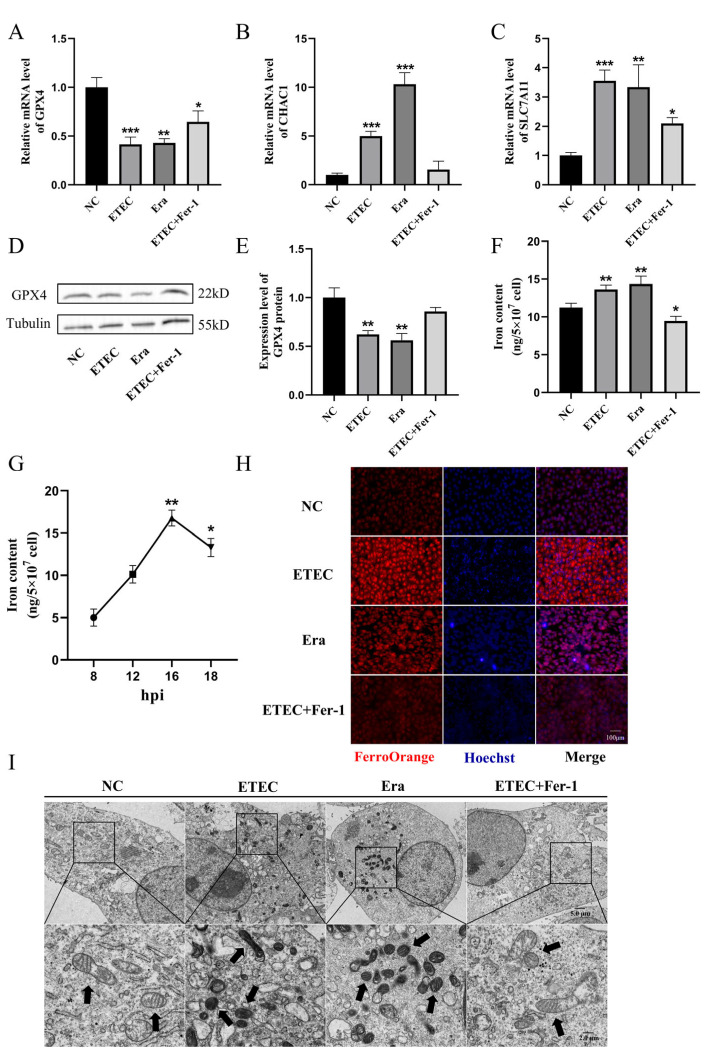
ETEC infection induces ferroptosis of IPEC-J2 cells. (**A**–**C**) GPX4, CHAC1, and SLC7A11 mRNA levels in wild-type IPEC-J2 cells (NC), or IPEC-J2 cells exposed to 400 MOI ETEC, 1 μmol/L Era, or ETEC + 10 μmol/L Fer-1. (**D**,**E**) The GPX4 protein expression in IPEC-J2 cells was revealed by Western blotting and relative quantification. (**F**) Comparison of intracellular iron in IPEC-J2 cells treated with ETEC, Era, or Fer-1. (**G**) Intracellular iron content of ETEC-infected IPEC-J2 cells at various time points post-ETEC infection (hpi). (**H**) Epifluorescence staining of intracellular Fe^2+^ (FerroOrange, red) in IPEC-J2 cells. Scale bar:100 μm. (**I**) Representative TEM images of organelle morphology in IPEC-J2 cells. Arrows indicate mitochondria. Scale bar:5 μm and 2 μm. All experiments were performed in triplicate. Data are expressed as the mean ± SD. * *p* <  0.05. ** *p* < 0.01. *** *p* < 0.001.

To further demonstrate the occurrence of ferroptosis upon ETEC infection, we detected the expression of ferroptosis-associated genes using qRT-PCR and Western blot. Consistent with Era treatment, ETEC infection decreased the expression of GPX4 (Figure 2A) and increased the mRNA levels of ChaC glutathione-specific gamma-glutamylcyclotransferase 1 (CHAC1) (Figure 2B) and solute carrier family 7 member 11 (SLC7A11) (Figure 2C). Conversely, Fer-1 co-treatment upregulated GPX4 expression, downregulated CHAC1 and SLC7A11 mRNA levels, suggesting that Fer-1 suppressed ETEC-induced ferroptosis in IPEC-J2 cells. Western blot was used to detect the key ferroptosis protein GPX4. The results showed that ETEC infection downregulated GPX4 expression, consistent with qRT-PCR results (Figure 2D,E). Similar to treatment with Era, ETEC infection significantly increased intracellular iron levels (Figure 2F). After ETEC infection, the intracellular iron content increased in a time-dependent manner, peaking at 16 h post-infection (hpi). (Figure 2G). The intracellular free iron levels were assessed by epifluorescence using the Fe^2+^-sensitive probe FerroOrange. Similar to Era treatment, ETEC infection resulted in higher Fe^2+^ levels, which were significantly reduced by the ferroptosis inhibitor Fer-1 (Figure 2H). The cell morphology was characterized using TEM. Cell membrane rupture, mitochondrial shrinkage, and cristae reduction were observed in IPEC-J2 cells infected with ETEC, consistent with the typical morphological features of ferroptosis induced by Era (Figure 2I). Fer-1 treatment blocked the ferroptosis morphology changes caused by ETEC infection. These results suggest that ETEC infection induces ferroptosis of IPEC-J2 cells via the SLC7A11/GPX4 pathway.

### 3.3. Nar Alleviates ETEC-Induced Oxidative Stress of IPEC-J2 Cells

**Figure 3:**
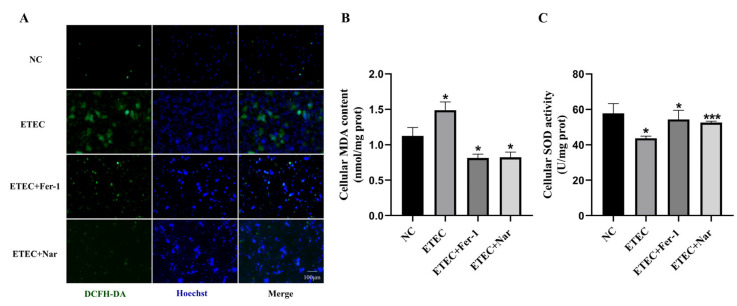
Nar alleviates ETEC-induced oxidative stress in IPEC-J2 cells. (**A**) Epifluorescence staining of intracellular ROS (DCFH-DA, green) in wild-type IPEC-J2 cells (NC), IPEC-J2 cells exposed to 400 MOI ETEC, ETEC+ 10 μmol/L Fer-1, or ETEC+ 50 μmol/L Nar. Nuclei were stained by Hoechst (blue). Scale bars:100 μm. (**B**,**C**) MDA and SOD accumulation in IPEC-J2 cells. All experiments were performed in triplicate. Data are expressed as the mean ± SD. * *p*  <  0.05. *** *p* <  0.001.

Using the ROS fluorescent probe DCFH-DA, it was found that Nar significantly reduced the green fluorescence intensity, thereby inhibiting the excessive accumulation of ROS in cells caused by ETEC infection, similar to that of adding the ferroptosis inhibitor Fer-1 (Figure 3A). Further testing with assay kits revealed that Nar significantly reduced MDA content and increased SOD activity (Figure 3B,C). These results suggest that Nar suppresses the oxidative stress induced by ETEC infection in IPEC-J2 cells and enhances their antioxidant capacity.

### 3.4. Nar Inhibits ETEC-Induced Ferroptosis of IPEC-J2 Cells

**Figure 4:**
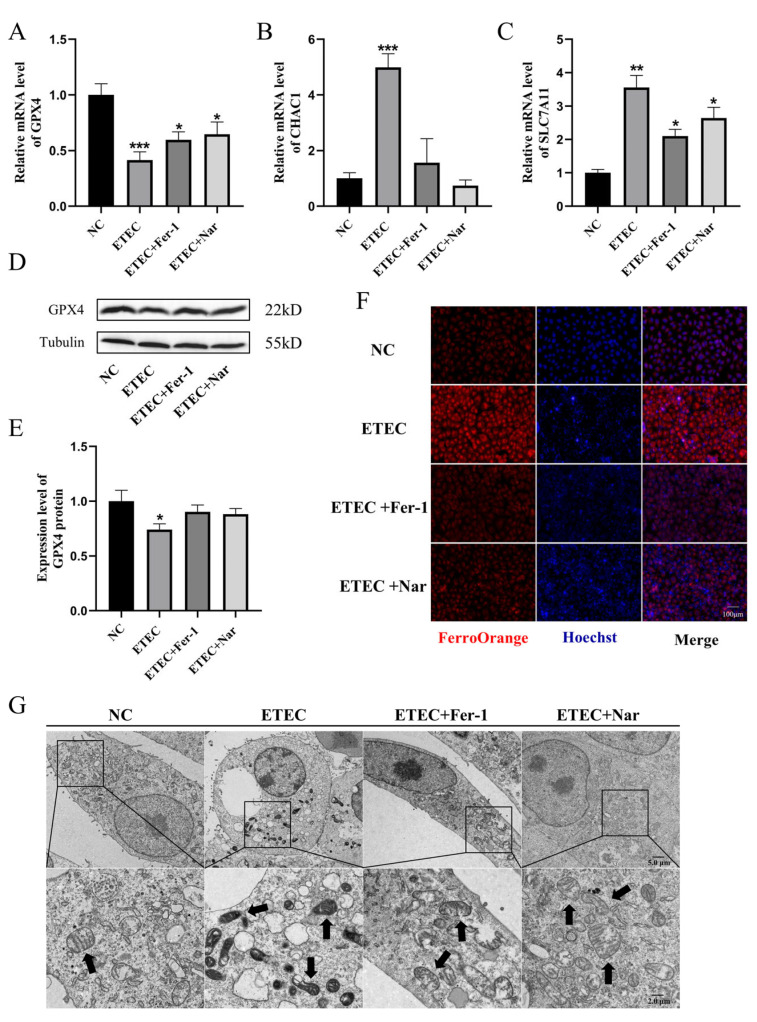
Nar inhibits ETEC-induced ferroptosis of IPEC-J2 cells. (**A**–**C**) The mRNA levels of GPX4, CHAC1, and SLC7A11 in wild-type IPEC-J2 cells (NC), cells infected with 400 MOI ETEC (ETEC), cells treated with ETEC and 10 μmol/L Fer-1 (ETEC + Fer-1), and cells treated with ETEC and 50 μmol/L Nar (ETEC+ Nar). (**D**,**E**) The GPX4 protein expression in IPEC-J2 cells was revealed by Western blotting and relative quantification. (**F**) Epifluorescence staining of intracellular Fe^2+^ (FerroOrange, red) in IPEC-J2 cells. Scale bar:100 μm. (**G**) Representative TEM images of organelle morphology in IPEC-J2 cells. Arrows indicate mitochondria. Scale bar: 5 μm and 2 μm. All experiments were performed in triplicate. Data are expressed as the mean ± SD. * *p* < 0.05. ** *p* < 0.01. *** *p* < 0.001.

As shown in Figure 4, Nar exhibited effects similar to those of the ferroptosis inhibitor Fer-1. Via qRT-PCR and Western blot characterization, Nar attenuated the downregulation of GPX4 (Figure 4A,D,E) and the upregulation of CHAC1 (Figure 4B) and SLC7A11 (Figure 4C) caused by ETEC infection, indicating that Nar inhibited the ETEC-induced ferroptosis in IPEC-J2 cells. The addition of Nar after ETEC infection significantly reduced the fluorescence intensity of the labile iron pool, indicating the attenuation of ferroptosis (Figure 4F). TEM characterization showed that Nar-treated cells could recover from the membrane rupture and mitochondrial shrinkage induced by ETEC, which was consistent with the effect of ferroptosis inhibitor Fer-1 (Figure 4G), indicating that Nar treatment played a role in alleviating ferroptosis of IPEC-J2 cells induced by ETEC infection.

### 3.5. Analysis of the Action Targets of Nar in the Treatment of Piglet Diarrhea by Network Pharmacology and Molecular Docking

**Figure 5:**
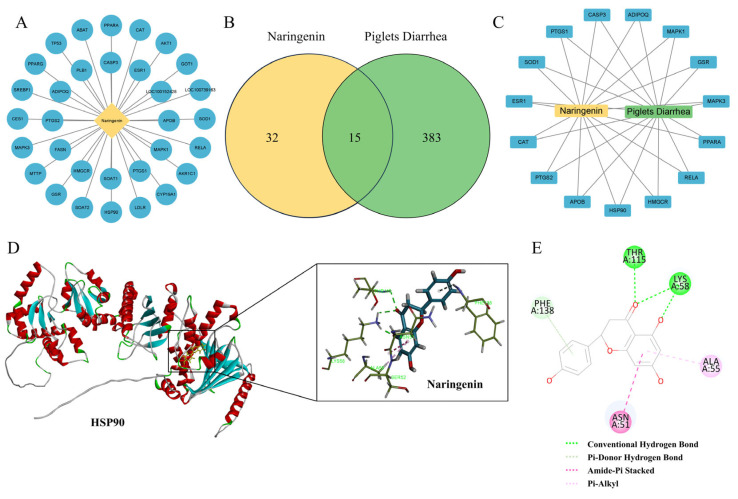
Analysis of the action targets of Nar in the treatment of piglet diarrhea by network pharmacology and molecular docking. (**A**) Targets related to naringenin. (**B**) Venn diagram of diarrhea and naringenin. (**C**) Diarrhea-naringenin target network. (**D**,**E**) Molecular docking analysis of naringenin and HSP90, which can interact by conventional hydrogen bonds, pi–donor hydrogen bonds, amide–pi stacking, and pi–alkyl interactions.

**Figure 6:**
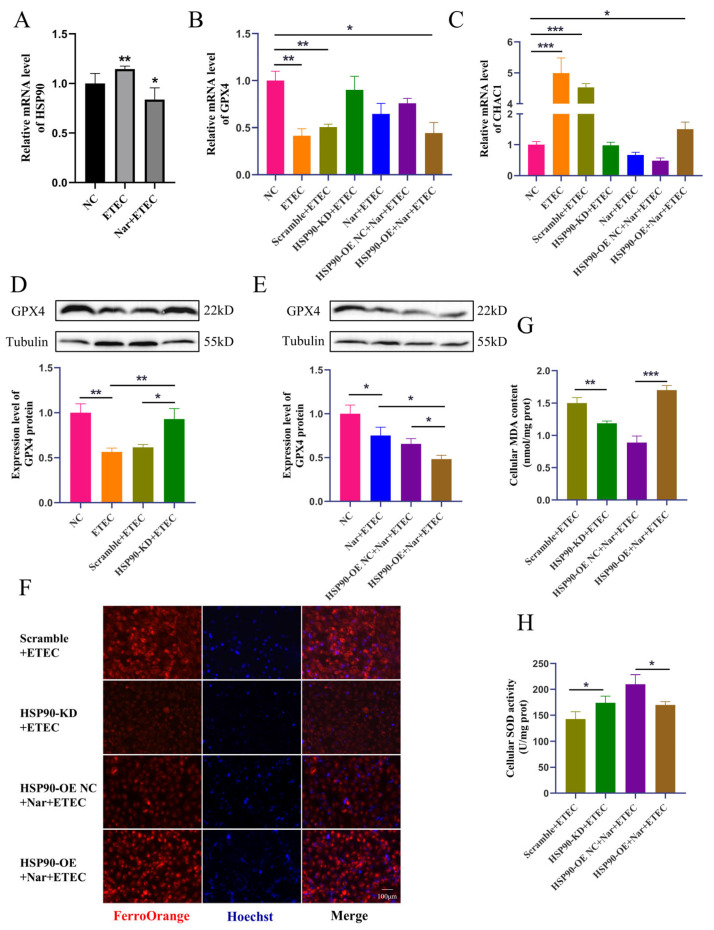
Nar inhibits ETEC-induced ferroptosis via targeting HSP90 in IPEC-J2 cells. (**A**) HSP90 mRNA levels in wild-type IPEC-J2 cells (NC), IPEC-J2 cells infected with 400 MOI ETEC (ETEC), or treated with 50 μmol/L Nar and ETEC (Nar + ETEC). (**B**,**C**) The mRNA expression levels of GPX4 and CHAC1 in wild-type IPEC-J2 cells (NC), cells infected with ETEC (ETEC), cells transfected with HSP90-KD siRNA scrambled control and infected with ETEC (Scramble + ETEC), cells transfected with HSP90-KD siRNA and infected with ETEC (HSP90-KD + ETEC), cells treated with Nar and ETEC (Nar + ETEC), cells transfected with empty vector and treated with Nar + ETEC (HSP90-OE NC + Nar + ETEC), and cells transfected with HSP90 overexpression vector and treated with Nar + ETEC (HSP90-OE + Nar + ETEC). For horizontal comparisons, different letters indicate significant differences, while the same letters represent insignificant differences. For horizontal comparisons, different letters indicate significant differences, while the same letters represent insignificant differences. (**D**,**E**) The GPX4 protein expression in IPEC-J2 cells of each treatment was revealed by Western blotting and relative quantification. (**F**) Epifluorescence staining of intracellular Fe^2+^ in IPEC-J2 cells for each treatment. The orange-red fluorescence emitted by the Fe^2+^ probe FerroOrange, while the blue fluorescence emitted by Hoechst detects DNA. Scale bar:100 μm. All experiments were performed in triplicate. (**G**,**H**) Intracellular MDA and SOD levels in IPEC-J2 cells of each treatment. Data are expressed as the mean ± SD. * *p* < 0.05. ** *p* < 0.01. *** *p* < 0.001.

To further investigate the molecular mechanism of Nar’s inhibition of ferroptosis in IPEC-J2 cells induced by ETEC infection, a network pharmacological approach was employed. This analysis identified 32 potential targets for Nar and 383 targets related to piglet diarrhea (Figure 5A,B). Using the Venn diagram online tool, we identified 15 common targets, which are considered potential therapeutic targets for Nar in the treatment of piglet diarrhea (Figure 5C). Analysis revealed that the therapeutic targets of Nar in piglets’ diarrhea include oxidative stress indicators such as PPARα, CAT and SOD (Figure 5C). Interestingly, heat-shock protein HSP90 is also a potential target of Nar, indicating that the inhibition of ferroptosis by Nar in IPEC-J2 cells may be related to the expression of HSP90. Molecular docking suggested that Nar and HSP90 might interact by conventional hydrogen bonds, pi–donor hydrogen bonds, amide–pi stacking, and pi–alkyl interactions, with a free energy of −115.16 kcal/mol, indicating Nar may suppress ferroptosis via the stable binding to HSP90 (Figure 6D,E).

### 3.6. Nar Inhibits ETEC-Induced Ferroptosis via Targeting HSP90 in IPEC-J2 Cells

ETEC infection upregulated the transcription of HSP90 in IPEC-J2 cells, while Nar treatment downregulated the mRNA level of HSP90, confirming that Nar targets and inhibits the upregulation of porcine HSP90 induced by ETEC infection (Figure 6A). Next, the protective mechanism of Nar on ETEC-induced ferroptosis was further explored in HSP90 knockdown IPEC-J2 cells (HSP90-KD) and HSP90 overexpression IPEC-J2 cells (HSP90-OE). The knockdown of HSP90 significantly increased the GPX4 expression levels, decreased CHAC1 mRNA levels, and reduced intracellular iron content post-ETEC infection, suggesting that the reduction in HSP90 blocked the occurrence of ferroptosis. Consistently, HSP90 knockdown also reduced cellular oxidative stress caused by ETEC infection, as evidenced by decreased MDA levels and increased SOD expression. Therefore, the ETEC infection-induced ferroptosis was dependent on the elevation of HSP90. On the contrary, overexpression of HSP90 diminished the effects of Nar on suppressing ETEC-induced ferroptosis. After ETEC infection, compared to Nar-treated IPEC-J2 wild-type cells, lower GPX4 expressions, higher CHAC1 levels, and increased iron accumulation were detected in Nar-treated HSP90-OE cells (Figure 6B–H). Nar also lost its anti-oxidant function in HSP90 OE cells post-ETEC infection, in which the MDA level was upregulated and the SOD level was downregulated. This indicates that the effect of Nar on alleviating ferroptosis and oxidative stress is mediated by the downregulation of HSP90 expression. Taken together, our findings suggest that ETEC infection upregulates HSP90 to induce ferroptosis of IPEC-J2 cells, while Nar downregulates HSP90 to suppress the ferroptosis induced by ETEC.

## 4. Discussion

As the first physical barrier of the gut, the epithelial cell layer plays a key role in intestinal barrier integrity. Excessive cell death in the intestinal epithelium disrupts barrier function, increases the permeability of the gut barrier, and causes the translocation of gut luminal contents, including commensal microbes and pathogens [17]. Numerous studies have reported that ETEC infection is associated with various types of programmed cell death of the porcine intestinal epithelial cells, such as apoptosis, necroptosis, and pyroptosis [18,19,20]. Both pyroptosis and necroptosis were associated with subsequent inflammation, such as secretion of TNF-α, IL-6, IL-8, and IL-1β. In vivo and in vitro studies also demonstrated that ETEC infection induces oxidative stress in IPEC-J2 cells, as well as the intestinal tissue of piglets [21]. Ferroptosis is accompanied by increased reactive oxygen species and lipid peroxidation, as well as massive accumulation of cellular iron [8]. Here, we report that the oxidative stress induced by ETEC is linked to another form of regulated cell death, ferroptosis.

GPX4, SLC7A11, and CHAC1 are identified as essential regulators of ferroptosis and redox homeostasis. SLC7A11 plays a role in transmembrane transportation of cystine for GSH biosynthesis, which is further involved in the conversion of lipid hydroperoxides into non-toxic lipid alcohols by the catalytic activity of GPX4, thereby protecting cells from ferroptosis [22]. Since SLC7A11 is the key component of cystine-glutamate antiporter (system Xc^-^), this is identified as the system Xc^-^/GSH/GPX4 axis, a ferroptosis core pathway [23,24,25]. CHAC1, a biomarker of ferroptosis, leads to GSH depletion and lipid peroxide accumulation as a γ-glutamyl cyclotransferase [26]. Our studies found that ETEC infection induced ferroptosis of IPEC-J2 cells by suppression of GPX4 and elevation of both SLC7A11 and CHAC1 transcription, suggesting that GSH regulation plays a vital role in this process. Many studies have revealed that ferroptosis activators, such as Era, inhibit SLC7A11-mediated cystine import. In response, SLC7A11 transcription is upregulated to compensate for the loss of function of system Xc^-^ [7,27]. Since ETEC infection is proven to be a ferroptosis activator in our research, we assume there is a similar adaptive response to ETEC in IPEC-J2 cells. Post-ETEC infection, more abundant SLC7A11 might be associated with the inhibition of system Xc^-^. Therefore, we highlight that ETEC infection induces ferroptosis in IPEC-J2 cells via the inhibition of the SLC7A11/GPX4 pathway.

Nar could exert its dual-action effects on both pathogens and host cells. We were unable to demonstrate that Nar could kill ETEC F41 in vitro at a concentration of 100 μmol/mL, which is the most concentrated solution we could dissolve due to the poor solubility of Nar. Although Wang et al. found the growth rate of *E. coli* decreased in a dose-dependent manner with increasing concentration of Nar (0 to 2.20 mM), suggesting Nar might exert antibacterial effects on *E. coli* [28]. They also revealed that *E. coli* is more resistant to Nar than *Staphylococcus aureus* (*S. aureus*). This resistance is likely due to the complex outer membrane of Gram-negative bacteria, which is less permeable to hydrophobic substances. Our study focused on the beneficial effects and the regulation mechanism of Nar on host cells rather than inhibiting pathogens. Numerous studies have confirmed that Nar has significant anti-inflammatory, anti-apoptotic, and antioxidant properties in vivo and in vitro [29,30]. Recent studies have shown that Nar inhibited the intestinal inflammation in mice with severe acute pancreatitis via the NLRP3 pathway [31]. Additionally, Nar also prevents the oxidative stress and ferroptosis of intestinal epithelial cells (IEC-6 cells) and a murine model of intestinal ischemia/reperfusion injury [14]. Wu et al. reported that Nar also protected intestinal health by regulating gut microbiota and elevating the expression of occludin and claudin-1 in the rat colon [30]. Taken altogether, Nar ameliorates intestinal damage in many diseases via the regulation of inflammation, oxidative homeostasis, tight junctions, and epithelial cell death.

Heat-shock protein (HSP) 90, a member of the chaperone family, plays a vital role in various organisms by participating in the folding, maturation, and activation of several client proteins, including kinases, transcription factors, or signaling molecules. In functionally deficient tissues, HSP90 is involved in multiple forms of programmed cell death, such as apoptosis, autophagy, necroptosis, and ferroptosis [32]. However, the mechanism by which HSP90 regulates ferroptosis remains controversial. Zhou et al. also found that an anti-tumor compound, Timosaponin AIII, promotes ferroptosis of non-small cell lung cancer cell lines by activating HSP90-mediated ubiquitination and degradation of GPX4 [33]. Another study revealed that overexpression of HSP90 promoted ferroptosis of the hippocampal-derived HT-22 cells with cerebral ischemia-reperfusion injury [34]. Therefore, HSP90 has emerged as a potential target for cancer treatment due to its role in mediating ferroptosis of cancer cells. On the contrary, it has also been suggested that inhibition of HSP90 can forcibly deplete glutathione (GSH) in tumor cells to weaken the antioxidant capacity of the cells, increase ROS content, decrease GPX4 expression, and therefore accelerate ferroptosis [35]. The role of HSP90 in ferroptosis varies depending on the cell type and disease context. Our study investigated the role of HSP90 in regulating ferroptosis in infectious diseases. ETEC infection upregulated the expression of HSP90 in IPEC-J2 cells, while the treatment of Nar could inhibit ETEC-induced ferroptosis by downregulating HSP90 expression in IPEC-J2 cells. Thus, Nar targets HSP90 to elevate GPX4 levels, thereby mitigating the oxidative damage in IPEC-J2 cells caused by ETEC infection.

## 5. Conclusions

**Figure 7:**
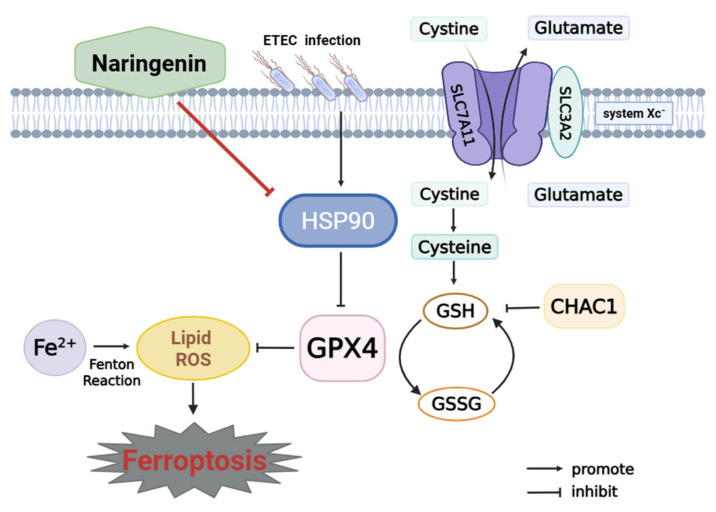
Mechanism of naringenin in inhibiting ETEC-induced ferroptosis in IPEC-J2 cells. Cystine-glutamate antiporter (system Xc^-^) is composed of SLC7A11 and SLC3A2. SLC7A11 plays a key role in transmembrane transportation of cystine for GSH biosynthesis, which is further involved in the conversion of lipid hydroperoxides into non-toxic lipid alcohols by the catalytic activity of GPX4. This is identified as the system Xc^-^/GPX4 pathway to prevent ferroptosis. CHAC1 leads to GSH depletion and lipid peroxide accumulation. Ferrous ion (Fe^2+^) accumulation, Fenton reaction, and inhibition of GPX4 cause lipid peroxidation to trigger ferroptosis. ETEC infection upregulates HSP90, which further inhibits the SLC7A11/GPX4 pathway, resulting in the activation of ferroptosis in IPEC-J2 cells. Naringenin inhibits HSP90 to attenuate ETEC-induced ferroptosis of IPEC-J2 cells.

This study reveals that ETEC infection causes ferroptosis of IPEC-J2 cells via the SLC7A11/GPX4 pathway. Nar, a natural flavonoid, targets host HSP90 to protect IPEC-J2 cells against ETEC infection-induced oxidative damage and ferroptosis (Figure 7). This work indicates that Nar could offer a novel approach for managing piglet diarrhea and safeguarding the intestinal health of young animals.

## Figures and Tables

**Table 1:** Primer sequences.

Gene Symbol	Accession Number	Primer Sequence (5′→3′)	Product Size/bp
GPX4 (pig)	NM_214407.1	F: CTGCTGGCTACAACGTCAAA R: AGTTCCATTTGTAGCATTTCCCA	135
CHAC1 (pig)	XM_021084905.1	F: TCCTTGAAGATTGTGAGGGCT R: CCTCCTTGGTGTCATAGCCA	126
SLC7A11 (pig)	XM_021101587.1	F: CCTGGGCAGGAGAAAGTTGT R: AGGACAAGGCTCCAAACAGT	193
GAPDH (pig)	NM_001206359.1	F: AACGTGTCGGTTGTGGATCT R: TCACAGGACACAACCTGGTC	140
HSP90 (pig)	NM_213973.2	F: AAGACCGGACCCTCACGATA R: AGGCATACTGCTCGTCATCG	231

## Data Availability

The data supporting the conclusions of this article are included within the article.

## Abbreviations

The following abbreviations are used in this manuscript:

CHAC1	ChaC glutathione-specific gamma-glutamylcyclotransferase 1
CTD	Comparative toxicogenomics database
ETEC	Enterotoxigenic *Escherichia coli*
Era	Erastin
Fer-1	Ferrostatin-1
FBS	Fetal bovine serum
GPX4	Glutathione peroxidase 4
GSH	Glutathione
HSP90	Heat-shock protein 90
HE	Hematoxylin and Eosin
IPEC-J2	Intestinal porcine epithelial cell line-J2
KD	knockdown
MDA	Malondialdehyde
MOI	Multiplicity of infection
Nar	Naringenin
PWD	Postweaning diarrhea
ROS	Reactive oxygen species
SOD	Superoxide dismutase
SLC7A11	Solute carrier family 7 member 11
SEM	Scanning electron microscopy
TEM	Transmission electron microscopy
ZO-1	Zonula occludens-1

## References

[1] Dubreuil J.D., Isaacson R.E., Schifferli D.M. (2016). Animal Enterotoxigenic *Escherichia coli*. EcoSal Plus.

[2] Liu G., Gu K., Wang F., Jia G., Zhao H., Chen X., Wu C., Zhang R., Tian G., Cai J. (2021). Tryptophan Ameliorates Barrier Integrity and Alleviates the Inflammatory Response to Enterotoxigenic *Escherichia coli* K88 Through the CaSR/Rac1/PLC-γ1 Signaling Pathway in Porcine Intestinal Epithelial Cells. Front. Immunol..

[3] Jin S., Xu H., Yang C., O K. (2024). Regulation of oxidative stress in the intestine of piglets after enterotoxigenic *Escherichia coli* (ETEC) infection. Biochim. Biophys. Acta (BBA)-Mol. Cell Res..

[4] Rao R.K., Basuroy S., Rao V.U., Karnaky K.J., Gupta A. (2002). Tyrosine phosphorylation and dissociation of occludin-ZO-1 and E-cadherin-beta-catenin complexes from the cytoskeleton by oxidative stress. Biochem. J..

[5] Zhou Y., Wang Q., Mark Evers B., Chung D.H. (2006). Oxidative stress-induced intestinal epithelial cell apoptosis is mediated by p38 MAPK. Biochem. Biophys. Res. Commun..

[6] Xia Y., Bin P., Liu S., Chen S., Yin J., Liu G., Tang Z., Ren W. (2018). Enterotoxigenic *Escherichia coli* infection promotes apoptosis in piglets. Microb. Pathog..

[7] Dixon S.J., Lemberg K.M., Lamprecht M.R., Skouta R., Zaitsev E.M., Gleason C.E., Patel D.N., Bauer A.J., Cantley A.M., Yang W.S. (2012). Ferroptosis: An Iron-Dependent Form of Nonapoptotic Cell Death. Cell.

[8] Jiang X., Stockwell B.R., Conrad M. (2021). Ferroptosis: Mechanisms, biology and role in disease. Nat. Rev. Mol. Cell Biol..

[9] Xu S., He Y., Lin L., Chen P., Chen M., Zhang S. (2021). The emerging role of ferroptosis in intestinal disease. Cell Death Dis..

[10] Zhang H., Xu Y., Zhang Z., You J., Yang Y., Li X. (2018). Protective immunity of a Multivalent Vaccine Candidate against piglet diarrhea caused by enterotoxigenic *Escherichia coli* (ETEC) in a pig model. Vaccine.

[11] Duan Q., Wu W., Pang S., Pan Z., Zhang W., Zhu G., Dudley E.G. (2020). Coimmunization with Two Enterotoxigenic *Escherichia coli* (ETEC) Fimbrial Multiepitope Fusion Antigens Induces the Production of Neutralizing Antibodies against Five ETEC Fimbriae (F4, F5, F6, F18, and F41). Appl. Environ. Microbiol..

[12] Motallebi M., Bhia M., Rajani H.F., Bhia I., Tabarraei H., Mohammadkhani N., Pereira-Silva M., Kasaii M.S., Nouri-Majd S., Mueller A.L. (2022). Naringenin: A potential flavonoid phytochemical for cancer therapy. Life Sci..

[13] Xu N., Liu S., Zhang Y., Chen Y., Zuo Y., Tan X., Liao B., Li P., Feng J. (2023). Oxidative stress signaling in the pathogenesis of diabetic cardiomyopathy and the potential therapeutic role of antioxidant naringenin. Redox Rep. Commun. Free. Radic. Res..

[14] Vikram A., Jayaprakasha G.K., Jesudhasan P.R., Pillai S.D., Patil B.S. (2010). Suppression of bacterial cell-cell signalling, biofilm formation and type III secretion system by citrus flavonoids. J. Appl. Microbiol..

[15] Hou M., Li X., Chen F., Tan Z., Han X., Liu J., Zhou J., Shi Y., Zhang J., Lv J. (2024). Naringenin alleviates intestinal ischemia/reperfusion injury by inhibiting ferroptosis via targeting YAP/STAT3 signaling axis. Phytomedicine: Int. J. Phytother. Phytopharm..

[16] Ling Z., Wang Z., Chen L., Mao J., Ma D., Han X., Tian L., Zhu Q., Lu G., Yan X. (2024). Naringenin Alleviates Radiation-Induced Intestinal Injury by Inhibiting TRPV6 in Mice. Mol. Nutr. Food Res..

[17] Patankar J.V., Becker C. (2020). Cell death in the gut epithelium and implications for chronic inflammation. Nat. reviews. Gastroenterol. Hepatol..

[18] Xiao K., Yang Y., Zhang Y., Lv Q., Huang F., Wang D., Zhao J., Liu Y. (2022). Long chain PUFA ameliorate ETEC-induced intestinal inflammation and cell injury by modulating pyroptosis and necroptosis signaling pathways in IPEC-1 cells—CORRIGENDUM. Br. J. Nutr..

[19] Xia Y., Chen S., Zhao Y., Chen S., Huang R., Zhu G., Yin Y., Ren W., Deng J. (2019). GABA attenuates ETEC-induced intestinal epithelial cell apoptosis involving GABA(A)R signaling and the AMPK-autophagy pathway. Food Funct..

[20] Xiao K., Zhou M., Lv Q., He P., Qin X., Wang D., Zhao J., Liu Y. (2023). Protocatechuic acid and quercetin attenuate ETEC-caused IPEC-1 cell inflammation and injury associated with inhibition of necroptosis and pyroptosis signaling pathways. J. Anim. Sci. Biotechnol..

[21] Wang Y., Zhang Z., Du M., Ji X., Liu X., Zhao C., Pang X., Jin E., Wen A., Li S. (2024). Berberine alleviates ETEC-induced intestinal inflammation and oxidative stress damage by optimizing intestinal microbial composition in a weaned piglet model. Front. Immunol..

[22] Ma T., Du J., Zhang Y., Wang Y., Wang B., Zhang T. (2022). GPX4-independent ferroptosis-a new strategy in disease’s therapy. Cell Death Discov..

[23] Lewerenz J., Hewett S.J., Huang Y., Lambros M., Gout P.W., Kalivas P.W., Massie A., Smolders I., Methner A., Pergande M. (2013). The cystine/glutamate antiporter system x(c)(-) in health and disease: From molecular mechanisms to novel therapeutic opportunities. Antioxid. Redox Signal..

[24] Yang W.S., SriRamaratnam R., Welsch M.E., Shimada K., Skouta R., Viswanathan V.S., Cheah J.H., Clemons P.A., Shamji A.F., Clish C.B. (2014). Regulation of ferroptotic cancer cell death by GPX4. Cell.

[25] Zhang H., Pan J., Huang S., Chen X., Chang A.C.Y., Wang C., Zhang J., Zhang H. (2024). Hydrogen sulfide protects cardiomyocytes from doxorubicin-induced ferroptosis through the SLC7A11/GSH/GPx4 pathway by Keap1 S-sulfhydration and Nrf2 activation. Redox Biol..

[26] Sun J., Ren H., Wang J., Xiao X., Zhu L., Wang Y., Yang L. (2024). CHAC1: A master regulator of oxidative stress and ferroptosis in human diseases and cancers. Front. Cell Dev. Biol..

[27] Zhang Y., Koppula P., Gan B. (2019). Regulation of H2A ubiquitination and SLC7A11 expression by BAP1 and PRC1. Cell Cycle.

[28] Wang L.H., Zeng X.A., Wang M.S., Brennan C.S., Gong D. (2018). Modification of membrane properties and fatty acids biosynthesis-related genes in *Escherichia coli* and Staphylococcus aureus: Implications for the antibacterial mechanism of naringenin. Biochim. Biophys. Acta (BBA)-Biomembr..

[29] Calis Z., Dasdelen D., Baltaci A.K., Mogulkoc R. (2022). Naringenin Prevents Inflammation, Apoptosis, and DNA Damage in Potassium Oxonate-Induced Hyperuricemia in Rat Liver Tissue: Roles of Cytochrome C, NF-κB, Caspase-3, and 8-Hydroxydeoxyguanosine. Metab. Syndr. Relat. Disord..

[30] Wu Y.X., Yang X.Y., Han B.S., Hu Y.Y., An T., Lv B.H., Lian J., Wang T.Y., Bao X.L., Gao L. (2022). Naringenin regulates gut microbiota and SIRT1/PGC-1ɑ signaling pathway in rats with letrozole-induced polycystic ovary syndrome. Biomed. Pharmacother. Biomed. Pharmacother..

[31] Yan X., Lin T., Zhu Q., Zhang Y., Song Z., Pan X. (2023). Naringenin protects against acute pancreatitis-associated intestinal injury by inhibiting NLRP3 inflammasome activation via AhR signaling. Front. Pharmacol..

[32] Peng C., Zhao F., Li H., Li L., Yang Y., Liu F. (2022). HSP90 mediates the connection of multiple programmed cell death in diseases. Cell Death Dis..

[33] Zhou C., Yu T., Zhu R., Lu J., Ouyang X., Zhang Z., Chen Q., Li J., Cui J., Jiang F. (2023). Timosaponin AIII promotes non-small-cell lung cancer ferroptosis through targeting and facilitating HSP90 mediated GPX4 ubiquitination and degradation. Int. J. Biol. Sci..

[34] Zhou Y., She R., Mei Z., Liu D., Ge J. (2024). Crosstalk between ferroptosis and necroptosis in cerebral ischemia/reperfusion injury and Naotaifang formula exerts neuroprotective effect via HSP90-GCN2-ATF4 pathway. Phytomedicine Int. J. Phytother. Phytopharm..

[35] Su X., Cao Y., Liu Y., Ouyang B., Ning B., Wang Y., Guo H., Pang Z., Shen S. (2021). Localized disruption of redox homeostasis boosting ferroptosis of tumor by hydrogel delivery system. Mater. Today Bio.

